# Supplementary material for: Feasibility and ethics of using data from the Scottish newborn blood spot archive for research
Source: Commun Med (Lond). 2022 Oct 6;2:126. doi: 10.1038/s43856-022-00189-2 (PMC9537278; doi:10.1038/s43856-022-00189-2)
Supplement: Supplementary file 1 — Supplementary Information [file 43856_2022_189_MOESM1_ESM.pdf]

Title

Feasibility and ethics of using data from the Scottish new-born blood spot archive for research.

Authors

Sarah Cunningham-Burley[1*#], Daniel L McCartney[2*], Archie Campbell[2*], Robin Flaig[3*], Clare EL Orange[4*], Carol Porteous[1], Mhairi Aitken[1], Ciaran Mulholland[5], Sara Davidson[5], Selena M McCafferty[4], Lee Murphy[6], Nicola Wrobel[6], Sarah McCafferty[6], Karen Wallace[7], David StClair[8], Shona Kerr[9], Caroline Hayward[9], Andrew M McIntosh[10], Cathie Sudlow[3], Riccardo E Marioni[2], Jill Pell[11], Zosia Miedzybrodzka[7], David J Porteous[2#]

Supplementary Information Tables

Supplementary Table 1: Mean (SD) DNA methylation levels at "rs" probes for each genotype group derived from SNP profiling.

SNP	Mean (SD) DNAm AA	Mean (SD) DNAm AB	Mean (SD) DNAm BB
rs877309	0.0251 (0.0129)	0.46 (0.0561)	0.863 (0.0296)
rs11249206	0.0311 (0.014)	0.407 (0.044)	0.86 (0.0198)
rs654498	0.448 (0.124)	0.474 (0.119)	0.516 (0.116)
rs715359	0.953 (0.0224)	0.459 (0.144)	0.0511 (0.00765)
rs6426327	0.065 (0.0219)	0.47 (0.0706)	-
rs6546473	0.0474 (0.0189)	0.443 (0.0682)	0.819 (0.0111)
rs264581	0.058 (0.0626)	0.386 (0.0513)	0.73 (0.123)
rs2125573	0.103 (0.0582)	0.49 (0.0872)	0.782 (0.0748)
rs9292570	0.426 (0.113)	0.494 (0.079)	0.668 (0.0689)
rs348937	0.611 (0.131)	0.333 (0.0509)	0.12 (0.0382)
rs9363764	0.799 (0.0446)	0.415 (0.121)	0.0611 (0.019)
rs10457834	0.741 (0.0703)	0.487 (0.0785)	0.109 (0.019)
rs6982811	0.103 (0.0691)	0.413 (0.0547)	0.785 (0.0553)
rs6471533	0.772 (0.282)	0.521 (0.207)	0.0524 (0.009)
rs472920	0.799 (0.0435)	0.468 (0.0898)	0.0985 (0.0211)
rs2385226	0.853 (0.18)	0.376 (0.0837)	0.0807 (0.0483)
rs4742386	0.835 (0.0587)	0.419 (0.117)	0.156 (0.181)
rs10796216	0.588 (0.146)	0.462 (0.0967)	0.413 (0.0837)
rs11034952	0.0963 (0.0349)	0.49 (0.123)	0.953 (0.00998)
rs2468330	0.0391 (0.0166)	0.43 (0.0719)	0.85 (0.046)
rs951295	0.0619 (0.00981)	0.571 (0.0576)	0.948 (0.0188)
rs2959823	0.749 (0.0864)	0.448 (0.0527)	0.195 (0.12)
rs1941955	0.83 (0.0448)	0.339 (0.0681)	0.0783 (0.0607)
rs2235751	0.0427 (0.0163)	0.385 (0.0558)	0.83 (0.0183)
rs1467387	0.0767 (0.0289)	0.412 (0.0847)	0.705 (-)
rs739259	0.0484 (0.0266)	0.405 (0.0618)	0.814 (0.0223)
rs2208123	0.543 (0.103)	0.468 (0.091)	0.428 (0.0577)

Supplementary Table 2: Citizens Jury terms of access

Q1: Who should have access to the Guthrie cards for research purposes?
Opinion: Access should be granted for the sole use, by a public or private sector organisation, who's clear rationale meets the stringent guidelines of the ethics committee.
Q2: For what purposes should research access be granted?
The primary purpose should be to advance medical research for the good of public health, through analysis of historical, current and future data.
Q3: What form of regulation and oversight would be required?
Opinion: An independent body, based on the Danish model (which includes a bio-centre management group, ethics committee and a data privacy committee), that is independent of government and data users, and able to issue sanctions for misuse.
Q4: Should any form of consent be sought and, if so, how would this work?
Opinion: There should be a universal [automatic] opt-in system, with the option to opt-out within one year or from the age of 16 The dead are automatically opted-in, with no consent necessary. Parents should be made aware of these conditions before their baby is born.
Q5: Whether and how the public might be involved in the process?
Opinion: Here there was consensus that once consent for research had been obtained there was no further need for public involvement, that parents should be well informed and that a website could communicate about research and outcomes.

Supplementary Information Methods

Citizens Jury

Supplementary Table 1 summarises the Jurors consensus views on terms of access to the new-born blood spots archive. This information was shared at the Stakeholders event and will inform the Public Consultation planned by Scottish Government Chief Scientist's Office.

New-born blood spot box contents

Supplementary Figure 1 illustrates how the Scottish new-born blood spot archive has been stored (boxes, sub-boxes and card bundles contents).

Supplementary Figure 2 illustrates representative cards (with personal details masked) to show a card that was not useable under the restrictions placed by the Caldicott Guardians on only approving samples be taken that would leave at least one blood spot intact (Supplementary Figure 2A) and another card where punches could be taken for testing (Supplementary Figure 2B).

DNA methylation analysis

To analyse whether all samples matched their reported sex, multiple dimensional scaling was performed on X chromosome probes, reducing DNAm data from 15316 CpGs to two coordinates for each sample. After plotting, there were two clusters corresponding to sex. Annotating each data point by reported sex revealed no mismatches (Supplementary Figure 3). Using "rs" probes on the EPIC array, estimated genotypes were checked against those generated at baseline using a SNP array. At these probes it is possible to observe a unimodal, bi-modal or tri-modal distribution depending on genotype (AA/AB/BB). DNAm levels at these probes were plotted against hard-called genotypes (i.e. SNP array). Tight clustering of DNAm levels was observed according to genotype (Supplementary Figure 4). Average methylation levels by genotype are summarised in Supplementary Table 2.

Supplementary Information Figures

Supplementary Figure 1 Newborn blood spot archive

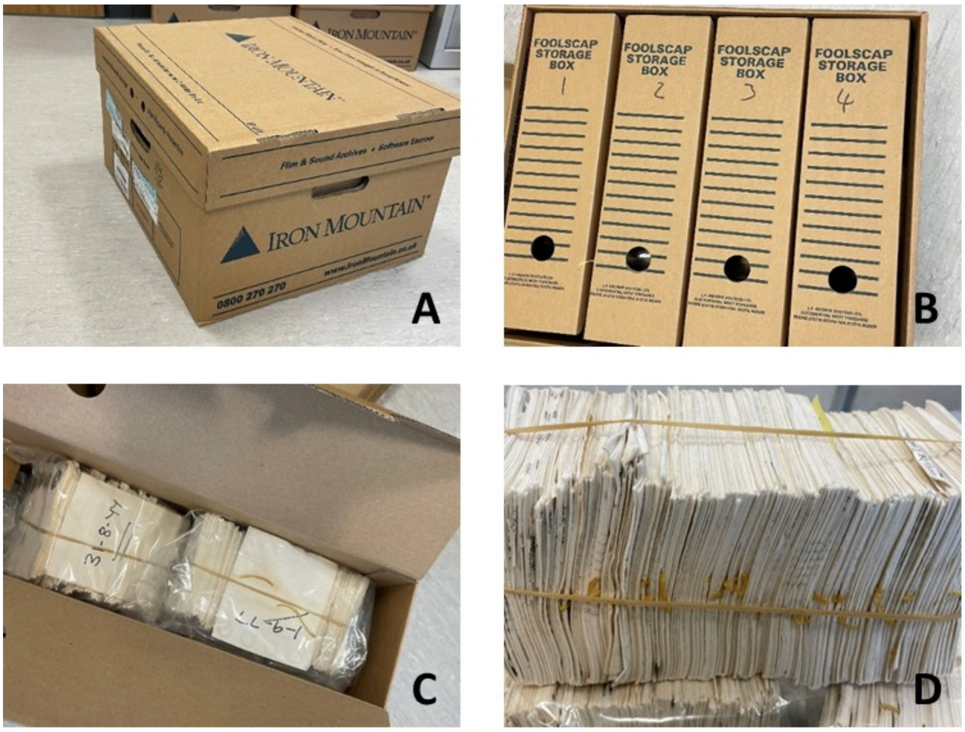

Legend: Newborn blood spot archives main box, barcoded (Panel A); sub-box, labelled (Panel B); sub-box, date-labelled bundles (Panel C); main box, unmarked bundles (Panel D).

Supplementary Figure 2 Newborn blood spot sampling

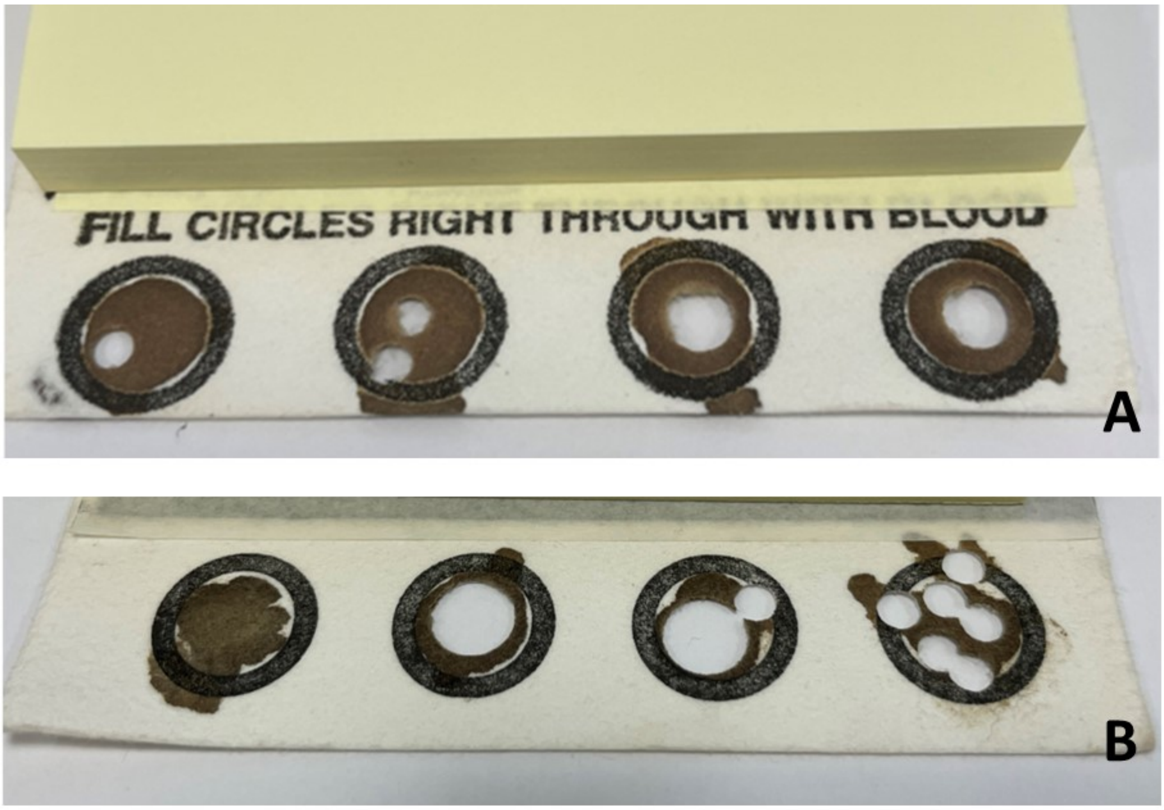

Legend: Panel A shows newborn blood spots not useable for feasibility study. All four spots had been sampled previously, excluding use in this feasibility study on account of the requirement set by the Caldicott Guardians that one spot remained untouched. Panel B shows newborn blood spots useable for feasibility study, with punches taken. The left hand spot had not been sampled, allowing seven 3mm punches to be taken for the feasibility study.

Supplementary Figure 3: Multidimensional scaling plot of X-chromosome probes coded by sample sex

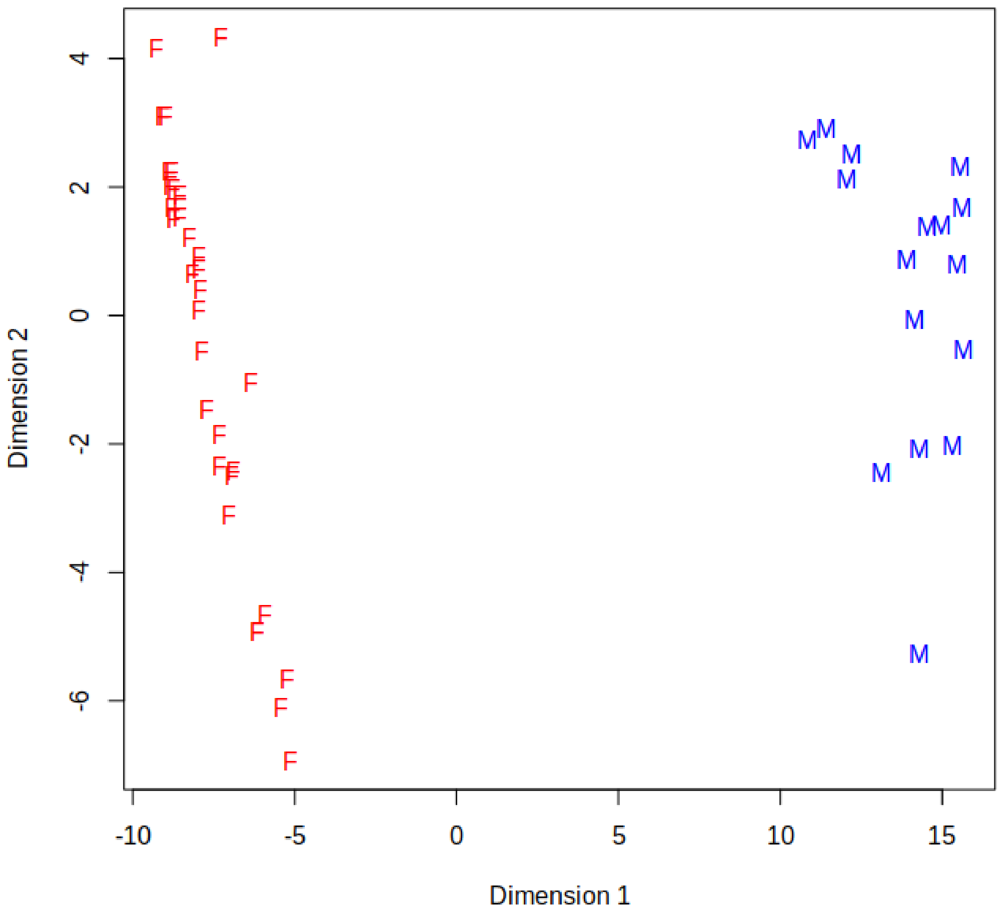

Supplementary Figure 4: DNAm Levels at “rs” probes with tight-clustering (y-axis) plotted against hard-called genotypes (x-axis)

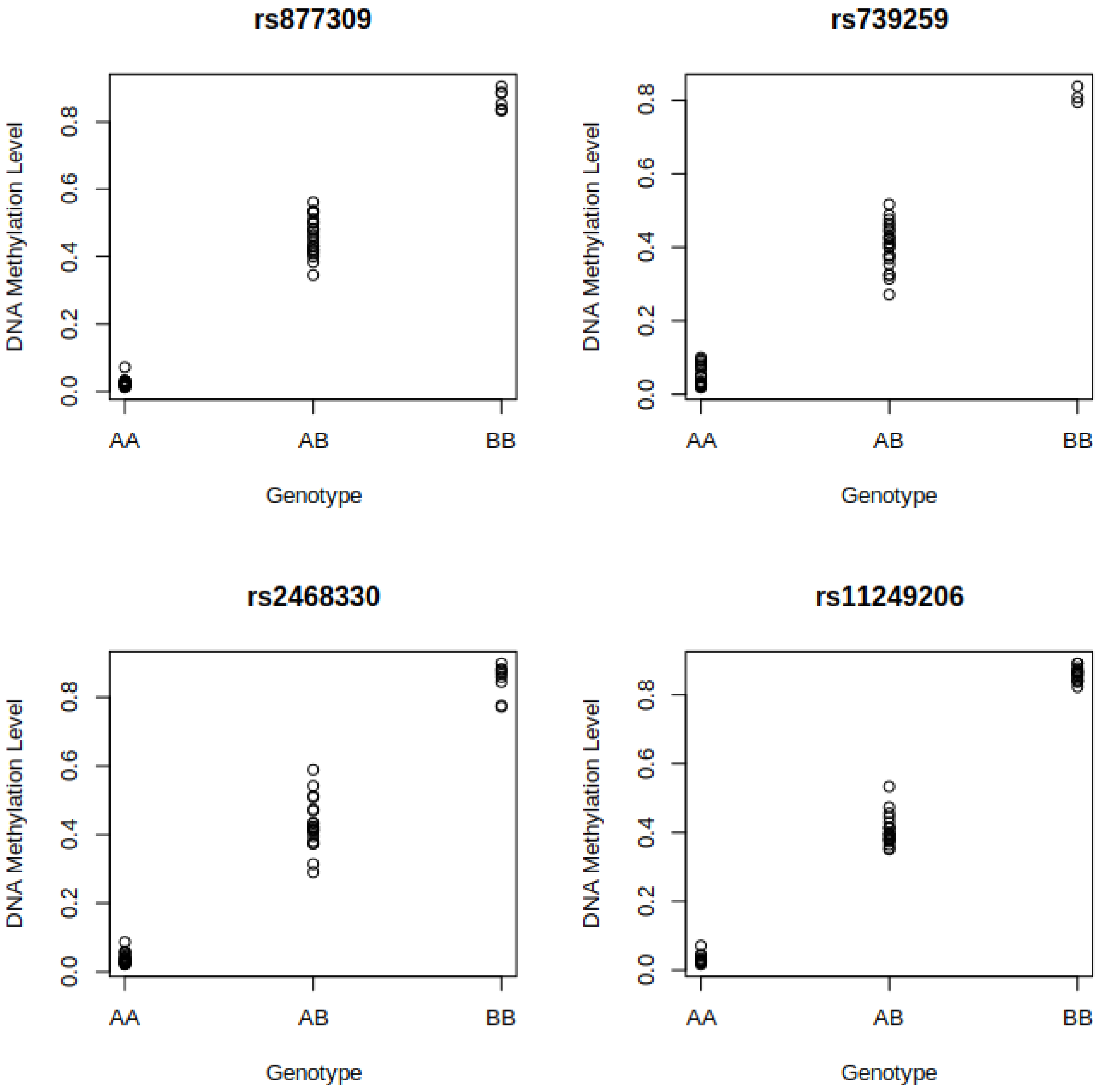

Supplementary Note 1

Use of Guthrie Cards for Research: Findings from a Citizens Jury, July 2017

Commissioned by Professors Sarah Cunningham-Burley and David J Porteous, University of Edinburgh

Funded by the Wellcome Trust Institute Science Support Fund to the University of Edinburgh

Conducted and report compiled by:

Dr Sara Davidson, Research Director sara.davidson@ipsos.com

Ciaran Mulholland, Associate Director ciaran.mulholland@ipsos.com

Ipsos MORI Scotland

This work was carried out in accordance with the requirements of the international quality standard for Market Research, ISO 20252:2012, and with the Ipsos MORI Terms and Conditions which can be found at http://www.ipsos-mori.com/terms.

Introduction

Background to the research

In most developed countries, screening of newborns is routinely undertaken to identify babies with serious health disorders in order that early treatment can be implemented and serious adverse health outcomes prevented. In Scotland, screening in the form of a heel prick blood spot test, or Guthrie Card, began in 1965 and has been routine for all newborns (roughly 60,000 each year) since the 1980s. The test currently screens for nine disorders for which early detection means early treatment and in the case of four conditions, the avoidance of severe neurological disorders. The blood spots are taken five to seven days after birth and held for a 12-month testing period. Between 1965 and 2003 consent to store the Guthrie Cards was presumed but since 2003 consent has been sought from parents to hold the Cards beyond the 12-month testing period.

The Guthrie Cards are, and continue to be, securely stored indefinitely for possible future clinical uses, however there is currently no legal framework governing *research* access to the collection. Blood spot collections around the world have and continue to be used for health-related research purposes, in countries such as Denmark, Australia and New Zealand. The legal position on research access in Scotland is unclear because the collection contains both personal information and tissue samples, which are treated differently by law. In 2009, the Chief Medical Officer for Scotland therefore placed a moratorium on research access to the Guthrie Card collection pending clarification of legal issues around consent, storage and access. The moratorium was extended to England, Wales and Northern Ireland in 2013.

Also in 2013, the Scottish Government published a report setting out the legal, ethical and social issues in relation to the use of the Guthrie Cards for research purposes. One recommendation made by the report was to conduct wider public engagement on the issue, to both inform the public about the potential arguments for and against research access to Guthrie Cards and to help involve the public in decisions about whether or not their blood sample should be used in such a way.

Accordingly, the University of Edinburgh commissioned Ipsos MORI to conduct research to explore public views on the question: *“Would research access to the Guthrie Card heel prick blood tests be in the public interest and, if so, under what conditions?”*

Methodology

The research was carried out using a citizens jury, which took place in Edinburgh over two day-long sittings on 17 and 24 June 2017. A citizens jury works in a similar way to a judicial jury and brings a small, representative group of citizens together to hear evidence, deliberate among themselves and reach a conclusion on the research question. A citizens jury is a recognised and proven method for enabling detailed, informed and productive deliberation among a diverse group of people who may not have previously engaged with the subject in question.

Rather than attempting to identify pre-existing attitudes to research access to the Guthrie Card blood tests, a concept which may not have been immediately relevant to participants nor fully understood, the citizens jury process allowed for the sharing of information and the development of considered responses over the course of the two sittings. Time dedicated to information sharing, the questioning of evidence, listening to the perspectives of others and finally negotiating consensus meant that participants were able to effectively deliberate on complex topics in considerable detail and produce informed recommendations.

As is common in citizens jury research, a gap of one week between the two ‘sittings’ was allowed, to give participants the opportunity to reflect on the information they received on day one, and, if they wished, share their thoughts with family and friends, or do some of their own research into the subject. This meant they returned on day two with more nuanced views as well as new questions and ideas to inform their subsequent deliberations on the research question.

Recruitment of participants

The target number of participants for the citizens jury was 16. Recruitment was undertaken by Ipsos MORI’s experienced in-house team of recruiters, using a face-to-face (door-to-door and in-street) free-find approach. The team was provided with a specially designed screener questionnaire to help them identify eligible participants. Quotas were set to ensure a representative pool of the public in terms of sex, age, working status, and social grade. Additional quotas were set to ensure sufficient representation of people with children under the age of five, and people who had, or whose had a family member with, a pre-existing medical condition. A further, attitudinal quota was set to ensure representation of people with varied levels of trust in public, private and third sector organisations[1], as previous research has found this to be a significant factor underpinning views on data sharing and use. Individuals who worked in market research, media, advertising, or journalism, and those who had attended a group discussion or event in the previous 12 months, were excluded from the research.

To allow for the possibility of some drop-out in advance of the citizens jury, an over-recruitment margin of c.25% was set, meaning 20 people were recruited. In the event, 19 participants attended day one of the jury, and 18 attended day two. The profile of those attending on day one is show in table 1.1.

Table 1.1: Profile of citizens jury participants

Category	Number of participants
Gender	
Male	11
Female	8
Age	
18-24	5
25-54	6
55+	8
Social grade	
ABC1	10
C2DE	9
Children under the age of 5	
Yes	4
No	15
Have/family have pre-existing medical condition	
Yes	10
No	9
Base: All who attended on day one (19)	

All participants were provided with a monetary incentive of £180 for taking part – £80 for attending the first sitting of the jury and £100 for attending the second.

Structure and content of the citizens jury

The citizens jury was structured around a discussion guide designed by Ipsos MORI in consultation with the University of Edinburgh. Day one of the jury was designed primarily to provide participants with information, or ‘evidence’ that would enable them to begin discussing the issues. Day two similarly included an information provision component but the major part of the day was devoted to participants’ own deliberations and, ultimately, to their arriving at a conclusion on the research question.

On both days, presenters – or ‘expert witnesses’ – provided participants with a range of information related to the subject of research access to Guthrie Cards. The presenters were identified and invited to attend by the University of Edinburgh.

The structure of each day is summarised in table 1.2.

Table 1.2: Structure of the citizens jury

Day one sessions	Purpose and content
Plenary session: Welcome and ice breaker	To welcome participants and outline the scope of the study.
Break-out groups: Views of health research	To explore unprompted attitudes towards health research, including: immediate reactions; positive and negative associations; and reasons for these views. Participants fed back key points in plenary.
Background information provision	To provide the first 'evidence' to aid deliberation.<br>- Professor Sarah Cunningham-Burley of the University of Edinburgh covered health research in general, background to the Guthrie Cards, and the current position regarding research access in Scotland<br>- Professor David Porteous, also of the University of Edinburgh described the Danish approach to using heel prick blood tests for research purposes.<br>Participants had the opportunity to reflect on the presentations and then ask questions.
Lunch break	
Expert witness testimony and Q&A	To provide further 'evidence'.<br><br>Four witness gave presentations at this stage:<br>- A leading academic researcher who gave an example of how Guthrie Cards could be used in research to understand health and health as we age<br>- A senior representative from NHS Scotland who described the measures currently taken by the NHS to protect people's privacy<br>- A leading academic clinical geneticist who discussed specific health conditions that could potentially be understood better though research using the Guthrie Cards<br>- A senior member of NHS Public Health and Health Policy and the Caldecott Guardians in Scotland, who explained how the NHS looks after data and weighs up and seeks to balance privacy and public benefit considerations<br>Participants had the opportunity to reflect on the presentations and ask questions of the expert witnesses.

Break-out groups with feedback in plenary: deliberation on expert testimony	To explore reaction to the presentations, including: any information that was surprising, anything that was concerning, general level of support or opposition to allowing research access to Guthrie Cards, any aspects they needed more information on to form a view.
Plenary session: Closing remarks	A summing up of the key messages from day one, description of next steps and completion of a questionnaire to provide participants with an opportunity to set out their current opinions in private.
Day two	Purpose and content
Plenary session: Welcome and recap	To welcome participants back, reflect on their current views on the research question and recap on information covered in day one.
Presentation of further evidence	To further aid deliberation.<br>The evidence provided comprised:<br>- a brief video clip of a news item from California covering arguments for and against collection and use of heel prick blood tests for research purposes<br>- a presentation from a senior NHS representative who explained how the potential for use of the Guthrie Cards in research might impact on parental uptake of the heel prick test, or otherwise jeopardise the NHS Scotland newborn blood spot archive.<br>- A senior representative from Genewatch UK who set out a range of possible risks associated with allowing research access to the Guthrie Cards.<br>Participants again had the opportunity to reflect on the presentations and ask questions of the expert witnesses.
Energiser exercise: ‘Speed dating’	To summarise current views and enable participants to hear each other’s views. Participants were asked to stand in pairs and each discuss what the most important consideration was for them at that point, before repeating this exercise in another pair.
Lunch break	
Group deliberation: identification of key considerations	To identify the broad considerations that participants felt were most important in assessing whether research access to the Guthrie Cards for research purposes was in the public interest.
Group deliberation: carousel exercise on key considerations	To enable participants to reflect on the key considerations in depth and arrive at conclusions in respect of each.
Group deliberation with feedback in plenary: refinement of views on the key question	To reach a conclusion on the research question.

Plenary session: Closing remarks	A summing up of the key messages from the citizens jury and completion of a questionnaire to gauge opinion at the individual level and compare this with the results from the day 1 questionnaire.

Materials

All the materials used in the citizens jury were designed by Ipsos MORI Scotland with input from the University of Edinburgh. These included topic guides which were used by moderators to facilitate each day, and questionnaires distributed to participants at the end of each day. Each of the expert witnesses prepared their own presentation, either in verbal in PowerPoint format.

Analysis

Both sitting of the citizens jury were audio-recorded (with participants' permission) and transcribed for analysis purposes. The transcripts and were then systematically analysed to identify the substantive themes that emerged in relation to each question in the discussion guide, along with key points and illustrative verbatim comments. This ensured that the analysis of the data was rigorous, balanced and accurate, and that key messages or concepts were brought out. It was also flexible enough to allow links and connections across different themes or sub-themes to be made, and for moments of interpretive insight and inspiration to be recorded.

Interpreting the data

Unlike survey research, qualitative social research does not aim to produce a quantifiable or generalisable summary of population attitudes, but to identify and explore the different issues and themes relating to the subject being researched. The assumption is that issues and themes affecting participants are a reflection of issues and themes in the wider population concerned. Although the extent to which they apply to the wider population, or specific sub-groups, cannot be quantified, the value of qualitative research is in identifying the range of different issues involved and the way in which these impact on people.

Deliberative approaches in particular, add value because of their ability to gain greater insight into what may lie behind people's opinions. They can also reveal how people's views can develop and change as they are given new information or through discussions with others on an issue. It should be noted, however, that, as participants' views are developed through deliberation, the outcomes cannot necessarily be taken to be representative of the views of the wider public who have not experienced the deliberative process.

It is worth nothing that the jury took place in the aftermath of a widely publicised data security breach in the NHS, in which 11 of Scotland's 14 health boards, as well as NHS trusts across England and Wales were affected by a ransomware attack. This may have impacted on the views participants offered, particularly in relation to issues of data protection and security.

Research findings

Context: attitudes towards health research in general

To provide context for participants' view on the potential use of Guthrie Cards in research, they were invited first to discuss and offer 'top of mind' thoughts on health research in general - including whether it was something they conceived of in mainly positive or negative terms, and whether they had any specific concerns or reservations about it.

Views were generally very positive, with participants pointing to the importance of research in advancing understanding and treatment of diseases and conditions, including diabetes, cancer and Alzheimer's Disease. Though voiced most keenly by people who themselves had or, who knew someone who had, a chronic disease or condition, this perspective was widely echoed and, indeed, over the course of the jury proved to be one of the strongest determinants of attitudes towards use of Guthrie Cards in research.

Most participants' support for health research extended to their being generally willing to participate in it and/or to see their medical records being used for this purpose. It was clear that many of them felt a deep moral obligation to do whatever they could to help both researchers investigating diseases conditions and treatments; and those whose lives the researchers are aiming to improve.

Despite this general level of positivity, and consistent with findings from other recent studies[2], participants commonly and spontaneously expressed reservations about health research involving commercial actors. Whereas they conceived of the public sector (and the NHS specifically) as motivated primarily by desire to promote the public good, they tended to describe the private sector, particularly the pharmaceutical industry, as "profit driven" and apt to prioritise financial gain above all else. A few people were keen to challenge this thinking, however, and stressed the importance of private sector investment in medical research and development; a perspective that appeared to gain traction as the jury progressed.

Another area of concern raised spontaneously by participants was data protection and security. They were keen to stress that any health data stored and used for research purposes must be safeguarded appropriately to minimise the risk of its falling into the wrong hands. To some extent these comments appeared to be influenced by a highly publicised ransomware attack on NHS computer systems across the UK that took place around a month prior to the first siting of the jury.

At the same time, however, there was a clear sense in which participants regarded health data as less sensitive than other types of information. They tended to explain this by commenting that the data did not contain any personal financial information that could be used to defraud data subjects in the event of a hacking incident. This echoed recent research exploring public attitudes to cross-sectoral data sharing for research purposes, in which banking and other financial information emerged as the only types of data that participants felt should be 'off limits'.

As is implicit in the foregoing discussion, participants' views on health research were evidently influenced by a combination of personal or proxy experiences and media coverage of related issues. There was a notable degree of ambivalence in respect of media coverage, however: On the one hand participants commented that the media had a tendency to highlight the exceptional rather than the typical – for example, in relation to data breaches – and thus to present a distorted picture of reality. On the other hand, they admitted to being heavily reluctant on the media for information about health-related developments, not least because they felt alternative channels of information were lacking.

The use of Guthrie Cards for research

Following the initial, general discussion about health research, participants heard from Sarah Cunningham-Burley, Professor of Medical and Family Sociology at the University of Edinburgh; and David Porteous, Professor and Chair of Human Molecular Genetics & Medicine, also at the University of Edinburgh. Sarah and David presented background factual information on:

- key considerations surrounding the use of patient information in health research (consent and control; openness and engagement; trust; who undertakes research; and the concept of the public interest)
- the newborn heel prick test and Guthrie Cards
- current clinical use of the Guthrie Cards and the moratorium on research use
- considerations surrounding the existence, continued storage and future uses of the Guthrie Cards for research purposes (legal complexities; the distinction between health records and tissue; issues of consent and anonymisation; questions of appropriate oversight; and the importance of public attitudes and engagement)

- how newborn blood spots have been stored and used for research purposes in Denmark, and the regulatory and ethical framework surrounding this practice Following the presentations, participants were given the opportunity to ask Sarah and David questions on points of fact. The jury was then adjourned for lunch, after which participants heard presentations from four of six expert witnesses who took part over the course of the two days (see Table for areas of expertise).

- Academic experts spoke about potential applications of Guthrie Card data in their respective fields of research, including examples of denial of access for research that might inform the risk of severe, progressive.
- Clinical experts set out the current steps taken by the NHS to preserve patients' privacy, in line with the requirements of the Data Protection Act plus background information on the Caldicott Guardians, senior people within the NHS with responsibility for protecting the confidentiality of patient information, outlining the seven Caldicott Principles, steps taken by the NHS to ensure that any information that could identify a patient is protected and only used when it is appropriate to do so. The importance of balancing privacy and public benefit considerations were highlighted.

Participants were given the chance to question the four witnesses, before taking part in facilitated discussions about the issues and considerations raised by the presentations.

Initial reactions to the expert testimony and the idea of using Guthrie Cards in research

Reactions to the presentations were very positive, with participants overwhelmingly expressing support for the use of Guthrie Cards in research. They felt there was a "clear public interest" case for allowing this, citing as reasons: potential advancements in the identification and/or prevention of disease; the development of new treatments; and, ultimately, improved population health. Some participants went further, commenting that it seemed wrong or "a waste" for the blood spots not to be used for research given

A related, though less common perspective was that using the blood spots for research would be considerably more cost- effective than the alternative of collecting "fresh" blood samples for every new study conducted. Asked whether they thought blood samples (and tissue generally) should be treated differently to other types of medical 'data' when it comes to research (for example, in terms of storage and usage) participants invariably said they did not .Indeed, several volunteered that they saw tissue as more anonymous and therefore less sensitive than medical records.

Despite participants' unanimous support at this point for the use of Guthrie Cards in research, they did raise some questions or concerns as to how the enterprise might work in practice. These centred on three main themes: data protection and security; control and oversight; and the possibility of 'creep' in allowed uses of the blood spots.

Data protection and security

Whereas some participants reported that the presentations– had left them feeling largely "reassured" about data protection and security in the NHS, others remained more cautious. They pointed out that, as with all organisations, there is a risk of the NHS mishandling data, which could result in information like the blood spots being "leaked" and used inappropriately.

Control and oversight

The emphasis on 'control' in the quotation immediately above, was a prominent feature of the discussion at this stage, reflecting, not only concerns about data protection and security, but about the number and types of organisations or individuals that might wish to access the blood spots, and the reasons they might wish to do so. There was broad agreement that "stringent guidelines" and governance would be required to ensure appropriate access and protect the public interest.

Questions were also raised about the extent to which data subjects themselves would be able to control whether and how their blood spots were used in research. For the most part, however, views on this issue were fairly relaxed and there appeared to be little appetite for a system of individual-based consent. Still, participants did wonder what would happen if a research study identified that a particular individual had a high risk of developing a serious disease or condition. There were differing views on what the appropriate protocol ought to be in this instance. Whereas some participants felt the researcher would have a moral obligation to ensure the individual was made aware of the risk, others commented that, if it was them, they "wouldn't want to know" if they had something wrong with them, particularly if it was a terminal or otherwise incurable illness.

Possible creep in uses of the blood spots

A small number of participants expressed concern that even with a robust system of governance in place, over time there could be an incremental increase in uses of the blood spots, beyond the types of health research envisaged in the presentations. There was specific mention of the data possibly being used by insurance companies to screen prospective customers, or by the criminal justice system to identify and prosecute offenders in cases where alternative forms of evidence are lacking. Some participants however, contended that the latter usage could be argued to be in the public interest and therefore appropriate.

Interim 'verdict' from the first sitting of the jury

At the end of the first sitting of the jury, participants were invited to complete a short questionnaire, designed to gauge (at an individual level) their current thinking on the potential use of Guthrie Cards in research. All reported that they believed the practice would be in the public interest, with half also saying they had no concerns about it at all, and the other half saying they had some minor concerns (Table 2.1).

Table 2.1: Attitudes towards the use of Guthrie Cards in research - close of day 1

At the moment, which of the following best describes your overall opinion on the use of Guthrie Card blood spots for research?	
Use of Guthrie Card blood spots for research is in the public interest and I have no concerns about it	10
Use of Guthrie Card blood spots for research is in the public interest but I have minor concerns about it	9

Use of Guthrie Card blood spots for research is in the public interest but I have major concerns about it	-
Use of Guthrie Card blood spots for research is not in the public interest	-
I don't know/am still making up my mind	-
Base: All day one participants (19)	

Those who said they had no concerns reiterated the view that there were no significant risks in using Guthrie Cards for research. They also commented that any concerns they may initially have had in relation to data protection issues had largely been assuaged by the witness presentations:

Those who said they had some minor concerns tended to reiterate the question of who would be given access to the data and/or to what end. They also commented that they would like to hear more about the "opposing point of view" on use of the Guthrie Cards for research, to ensure they were arriving at a verdict based on all of the most important considerations, both positive and negative.

Introduction to day two

On day two of the jury, participants were provided with further information in the form of a short video clip and presentations by two final witnesses. Reflecting participants' feedback from day one, the information intentionally included cautionary perspectives and considerations surrounding use of the Guthrie Cards for research.

The short video clip was a news item from California, which described the process of collecting newborn blood spots in the state, the reasons for this (i.e. the early detection and treatment of particular conditions) and other background information, such as how the samples were stored and used, including in relation to research. It also featured interviews with two parents with opposing perspectives on use of the blood spots; one who was in favour of the samples being used for research (for reasons similar to those set out in the previous chapter) and one who had concerns about this practice (including the fact that the police and private companies could buy the data to carry out investigations and research without parental consent). The news item also included an interview with a clinician who noted that there was no guarantee that blood samples could not be linked to personal information by someone with the requisite skills. He nonetheless felt that the samples presented a potentially invaluable research resource.

Participants then heard from an NHS Consultant in Public Health, who discussed current clinical uses of the Guthrie Cards and associated safeguards. Any future system for allowing research access to Guthrie Cards would need to be very carefully managed so as not to impact on parental uptake of the heel prick test, or otherwise jeopardise the very existence of the NHS Scotland newborn blood spot archive. Reference was made to the situation in Texas, where 5 million blood samples were destroyed following a lawsuit which found that parental consent for storage of the samples had not been obtained.

The final witness cautioned against allowing research access to the Guthrie Cards on the grounds that 'researchers' could include companies such as Google, which may, for example, seek to use the data to target the marketing of drugs and treatments at individuals at risk of developing particular diseases or conditions. In discussing this point, the concept of 'false positives' was introduced and the fact that unregulated genetic tests on blood samples can lead to people being

wrongly identified as being at risk, and thus to being offered unnecessary treatments. The question of the robustness of current data security and privacy practices in the NHS was raised, citing examples of recent data breaches. The importance of fully informed consent for any research using Guthrie Cards was emphasised, including data subjects being told who is funding the research and whether data is being handed over to private companies.

Following the presentations, and as on day one, participants were given the chance to put questions to the witnesses, before taking part in follow up facilitated discussions.

Initial reaction to the information

Despite the more cautionary tone of some of the information provided on day two, participants remained generally in favour of research access to the Guthrie Cards. Reasons given were very similar to those that emerged on day one, with reference again being made to potential public benefits in the form of advances in the detection, diagnosis and treatment of conditions. There was a general sense in which participants felt that arguments against research use of Guthrie Cards, though worthy of careful consideration, did not outweigh the potential benefits.

The information provided on day two also served to reinforce some of the questions and concerns raised on day one, particularly who would be able to access the Guthrie Cards for research. Concern about the possibility of research access being granted to commercial organisations, such as Google or pharmaceutical companies, was more pronounced than on day one, with participants speculating on how such organisations might use the data, and how this might be controlled.

Reflecting these concerns, participants reiterated the view that if research access is to be allowed, a robust system should be put in place to assess access requests and monitor research use, and that such a system should ensure use of the data is in the public interest. It was suggested that the system could take the form of peer review of research applications, as is common practice for existing medical research, or review by an ethics committee, as occurs in Denmark.

Thus, following the information provision session at the outset of day two, and though participants remained generally positive in their views, they began to move away from broad ‘for’ and ‘against’ arguments towards more nuanced discussions of the issues. Such discussions formed the basis of the main deliberative stage of the jury.

Detailed deliberation on the research question

Considerations against which research access to Guthrie Cards should be assessed

As a first step to reaching their final verdict, Jurors were asked to identify and agree on considerations they felt were key in deciding whether or not research access to the Guthrie Cards was in the public interest – taking into account the information they had been presented with at both sittings of the jury, the questions and concerns this had raised, and discussions they had had with their fellow Jurors and/or with their own friends and family during the week between the two sittings.

Five key considerations were identified, most of which had already been discussed to some extent over the course of the two days. These were:

- who should have access to the Guthrie Cards for research purposes?
- for what purposes should research access be granted?
- what form of regulation and oversight would be required?
- whether any form of consent should be sought and, if so, how this should work?
- whether and how the public might be involved the process?

Having identified these broad considerations, participants were invited to discuss them in depth (both in small groups and as a fully 'jury'), with a view to arriving at conclusions in respect of each.

Who should have access to the Guthrie Cards for research purposes?

As we have seen, the question of 'who should have access?' recurred throughout the jury process; it was raised early and spontaneously on day one in relation to health research generally, and it came up repeatedly in the group deliberations about use of Guthrie Cards in research. Now, at this later stage in the deliberations, the relative trustworthiness of different types of organisation that might wish to have access was discussed, with certain organisations viewed more positively than others in this regard than others. Jurors were generally positive about the NHS, universities or health-related charities being granted access, as they felt these organisations were likely to carry out research aimed at improving public health. In contrast and echoing views expressed on day one, some participants were cautious about granting access to private companies, such as Google and pharmaceutical companies. This was linked to belief that such organisations might use the information for reasons other than the public good; for example, profit generation, which may in fact be to the public detriment. Concern was expressed over a general perceived lack of regulation and control over such companies, which it was felt might be reflected in a lack of control over their use of the data.

Other participants, however, again suggested that private drug companies may also carry out research intended to benefit the health of the public, and that making profit and improving health may not be mutually exclusive.

Notwithstanding these mixed views on pharmaceutical companies, as the deliberations progressed, the focus moved away from individual organisations or sectors towards a wider discussion around the motives behind any research that is conducted and how these might be discerned and assessed. In other words, it became apparent that the question of *who* should have access to the Guthrie Cards was inextricably linked in participants' minds with the *purpose* of the research - and that the latter consideration was in fact the more salient for them. Indeed, Jurors went on to suggest that requests for access to the data should be judged on the rationale put forward for access, rather than on the organisation making the request.

However, some caution remained around how much control could be exercised over organisations once research access has been granted. Jurors spontaneously raised the hypothetical scenario of an organisation being granted access, but then passing the information on to another part of their organisation or to a third party that might use it for a different purpose to that originally intended. In light of such concerns, it was suggested that a condition should be placed on research access, to the effect that the information can be used solely for the purpose specified in the access request. In cases where this condition was contravened, it was felt that an appropriate penalty should be imposed such as a fine or a ban on any future research access.

As for all of the conditions they identified earlier in the discussion, Jurors were invited to condense their deliberations about who should have access to the Guthrie Cards for research purposes down to a single statement summarising their position. The statement the Jurors came up with was: *"Access should be granted for the sole use, by a public or private sector organisation, who's clear rationale meets the stringent guidelines of the ethics committee".*

For what purposes should research access be granted?

There was general agreement that research access should only be allowed for studies concerned with promoting the "greater good", which as already noted, tended to be conceived of primarily in public health terms; for example, "predicting future [health] issues and preventative measures" and "tracking changes across generation".

At the same time, and echoing reactions to the information provided on day one, there was suggestion that, in exceptional circumstances, blood spots could be used to assist with criminal

investigations in cases where there was a lack of alternative, viable evidence. In contrast, participants were unanimous that no access should be granted for any form of marketing activity, including targeted sales by insurance companies, or to enable employers to check up on their employees' health.

Jurors were keen that, in the event of research access to Guthrie Cards being granted, researchers should be able to draw on past, current and future data – in other words data from those who are alive at the time the research is commissioned, those who have died, and those yet to be born - to allow the greatest scope for insights. The issue of whether and how consent should be sought from these groups is explored in more detail in the section on consent below.

Ultimately, much of the discussion on the purpose of the research came back to consideration of the process for deciding whether or not a proposed study was in the public interest. Participants reiterated their view that a "system" should be put in place for reviewing, and evaluating research access proposals.

Concluding their deliberations on appropriate uses of the data, the Jurors summarised their position thus: *The primary purpose should be to advance medical research for the good of public health, through analysis of historical, current and future data.*

What form of regulation and oversight would be required?

As already noted, appropriate regulation and oversight of any research access to the Guthrie Cards was seen as vital. Views on this theme seemed to be very much influenced by the Danish 'model' (discussed by the presenters on day one), wherein all proposals to use newborn blood spots for research purposes are subject to the approval of a research ethics committee. Participants commonly suggested that Scotland should follow a similar approach to avoid 'reinventing the wheel.'

There was specific suggestion of appointing a regulatory body charged with making all ethical judgements surrounding use of the Cards for research, as well as decisions on data privacy issues and management of the facility where the Guthrie Cards are stored. Further, it was suggested that the body should consist of individuals with relevant experience and knowledge, such as experts from the fields of medicine and health research, but should be independent of government and of any data users, so that it remains impartial in its decision making and to minimise the likelihood of individuals with a vested interest in particular research studies having too much influence on decisions.

It was agreed that the body should have the power to issue sanctions against those who misuse the data or who contravene the conditions on which they have been granted access. There was also a consensus that it should be as "transparent" as possible in its decision making to help ensure public confidence in the process.

The group summarised their views on regulation and oversight by stating that there should be: *an independent body, based on the Danish model (which includes a bio-centre management group, ethics committee and a data privacy committee), that is independent of government and data users, and able to issue sanctions for misuse.*

Whether any form of consent should be sought and, if so, how this should work?

Jurors were encouraged to consider various facets of consent, including whether it was required at all; and, if so: from whom; whether an opt-in or opt-out system was most appropriate; and the stage at which any consent should be sought. They were also asked to consider whether researchers should be allowed to access the samples of people who have died or only those who are living and therefore in a position to give consent.

Jurors agreed that it was important some form of consent be obtained on behalf of, or from, data subjects in order that they have a degree of choice and control over whether or not their Guthrie Card is used for research. They suggested that parental consent should be sought following the birth of a child but that, once the child turns 16, s/he should be able to review and overturn the decision. It was suggested that parents to be should be provided with information about the Guthrie Cards' potential research uses *before* their child is born, in order that they have adequate time to digest and assess the implications. There was specific suggestion that the information could be included within the 'blue notes' given to expectant parents as a record of their pre-natal health progress. Providing the information before birth was seen as preferable to doing so immediately afterwards, when new mothers are already presented with a wide range of information, and also may not be in the correct frame of mind to consider and decide on issues of consent.

There was a preference for an opt out rather than opt in system of consent to avoid potential administrative complexities involved in attempting to obtain consent each time a request for research access to the Guthrie Cards is made.

Jurors noted that, under such a system, there would be no need to obtain consent from people who have died, thus further helping to mimimise administrative complexities.

In the case of living people for whom an existing blood spot is held, it was suggested that they should be treated on a similar basis as future cohorts in terms of being given an opportunity to opt-out. There was suggestion that this could be done via an awareness raising campaign, consisting of notices or advertisements in a range of media channels.

Discussion of consent also led to consideration of whether the blood spots should be destroyed where consent for research use is refused. Opinion was split on this issue. Those in favour of destruction felt that if individuals are being given the choice to opt-out of research, they should simultaneously be given the choice to have their record destroyed as a guarantee that it will not be used at any point in the future. Similarly, there was suggestion that people with particular religious or other fundamental beliefs that might preclude their blood being kept and used for research, should have some means of ensuring that this does not happen. Those who opposed destruction, on the other hand, felt that the opt- out system was enough of a guarantee that the records would not be used for research. They also commented that destruction would deny individuals the right to choose for themselves at age sixteen what should happen to their data.

In summarising their position on consent, Jurors concluded that: *there should be a universal [automatic] opt-in system, with the option to opt-out within one year or from the age of 16 The dead are automatically opted-in, with no consent necessary. Parents should be made aware of these conditions before their baby is born.*

Public involvement

Two facets of public involvement were raised by participants: firstly, whether or not the public should have an ongoing role in decision-making on research carried out; and, secondly, the extent to which the public should be kept informed about any research that does take place.

On the first of these questions, there was a consensus that once consent for research has been obtained, there would be no further need for the public to have a say on research carried out. Indeed, some participants went further, contending that any ongoing public involvement beyond the consent stage could serve to disrupt the research process, as it may prompt individuals to change their minds about consent they had previously given and try to reverse.

In terms of keeping the public informed, as outlined above participants suggested that new parents should be provided with as much information as necessary before their child is born to enable them to decide on whether or not to opt out. Beyond this, they felt that a central webpage could be set up, detailing the types of research being carried out using the blood spots and, later, what the

outcome of the research have been. It was felt that the information contained on the website should be open and honest in these sense of sharing both success stories from research, as well as any less positive outcomes.

It was also suggested that, alongside the website, data users should be able to publicise how they are using Guthrie Cards as they see fit and using their own channels.

Overall verdict of the Citizens Jury

In summary,and based on everything they had heard and discussed over the two sittings of the jury, participants unanimously agreed that research access to the Guthrie Cards was in the public interest. As table 2.2 shows, the distribution of opinion recorded in the end of event questionnaire, was little changed on that recorded at the close of day one, with approximately half of participants saying they had no concerns, and a similar proportion saying they had minor concerns. Only one participant said they had major concerns.

While Jurors agreed that research access to Guthrie Cards was in the public interest, this position was subject to the following conditions:

- The ultimate purpose of the research should be to advance medical research in ways that could potentially benefit public health.
- Any organisation requesting access to the data should provide a clear rationale for their research, which should be approved by an ethics committee. Access should be granted solely for the use outlined in their research request.
- There should be appropriate regulation and oversight of the process by an independent body - that includes an ethics committee - with the authority to issue sanctions for misuse.
- Consent should be based on an opt-out system, with the option to opt-out within one year of the child being born, and again when the child turns sixteen.
- Mechanisms should be put in place (in the form of a central website) that enable members of the public to check on the types of research being undertaken with Guthrie Card data and the outcomes.

Table 2.2: Attitudes towards the use of Guthrie Cards in research – day 1 and day 2

Which of the following now best describes your overall opinion on the use of Guthrie Card blood spots for research?		
	Day one	Day two
Use of Guthrie Card blood spots for research is in the public interest and I have no concerns about it	10	8
Use of Guthrie Card blood spots for research is in the public interest but I have minor concerns about it	9	9
Use of Guthrie Card blood spots for research is in the public interest but I have major concerns about it	-	1
Use of Guthrie Card blood spots for research is not in the public interest	-	-
I don’t know/am still making up my mind	-	-
Base: All day two participants	*19*	*18*

Conclusions and recommendations

The citizens jury research identified a strong level of public support for research access to the Guthrie Cards blood spots. In part, this support reflected participants' pre-existing belief that health research generally was crucial in advancing understanding and treatment of diseases and conditions in today's society – a belief that appeared only to strengthen over the course of the two days. Guthrie Cards were regarded as just one more valuable form of health data, along with tissues samples generally and medical records, that researchers should be able to draw on to inform their investigations and ultimately to promote improved population health.

Notwithstanding this positive position, participants did spontaneously raise some questions and concerns about research access to the data. These were inter-related and centred around:

- data security, and a perceived risk of the information being hacked or leaked and used inappropriately (though information provided at the jury about data privacy and protection arrangements in the NHS did appear to assuage these concerns to a significant degree).
- commercial organisations potentially being granted access to the data and using it for reasons other the public good; in particular for marketing or profit generation.
- the extent which the process would be regulated and overseen to avoid the sorts of issues outlined above and to instil public confidence in the process.

Reflecting these areas of concern, the jury's support for research access to the Guthrie Cards was contingent upon a number of key conditions being met; namely:

- the purpose of the research being to advance medical research in ways that could potentially benefit public health.
- any organisation requesting access to the data providing a clear rationale for its research, which should be approved by an ethics committee. Access should be granted solely for the use outlined in their research request.
- there being appropriate regulation and oversight of the process by an independent body – that includes an ethics committee - with the authority to issue sanctions for misuse.
- there being an opt-out system of consent, with the option to opt-out within one year of the child being born, and again when the child turns sixteen.
- mechanisms being in place (in the form of a central website) that enable members of the public to check on the types of research being undertaken with Guthrie Card data and the outcomes.

Limitations of the research and potential future work

The citizens jury produced very clear findings and a definitive answer to the research question. However, when considering the results, it is prudent to bear in mind certain limitations of the study.

Firstly, the expert witnesses played an important and influential role in the jury process. It was from the witnesses that participants received most of their information about Guthrie Cards (several had been unaware of the Cards prior to attending the jury) and heard arguments in favour of and against using the blood spots for research. In light of this influential role, and as in any citizens jury, the ideal scenario would have been for the witness testimony presented each day to reflect a roughly equal balance of positive and negative perspectives on the issues. While both types of perspective were presented over the course of the jury, information on day one was more weighted towards arguments in favour of research access than against. This may have played a role in

shaping participants' views in a positive direction. However, and as already noted, most participants did come to the jury positively pre-disposed towards health research, which may suggest that they would have supported research access to the Guthrie Cards whatever the order and balance of information presented.

Secondly, in recruiting the jury, no attempt was made to purposively sample individuals with particular religious or other fundamental beliefs that might have had a bearing on their reaction to the idea of Guthrie Cards being used in research. In any future research, it may useful to target such people, perhaps through in-depth interviews or focus groups, to ensure the full range of perspectives is considered.

While the citizens jury delivered an unequivocal verdict on the central question it cannot, by virtue of its scale, be presented as a robust representation of the wider public's views on the issue. There would therefore be merit in attempting to assess the extent to which the jury's verdict and associated considerations are reflected among the wider population. This would best be done through quantitative methods and, in particular, techniques that allow for the exploration of multiple considerations and trade-offs, such as discrete choice or 'Max Diff' experiments.

Finally, the readiness and ability of participant to engage with the subject of research access to Guthrie Cards, deliberate on it and arrive at a verdict suggest there is merit in exploring opportunities for ongoing public engagement in this area. More generally it provides a strong case for wider use of deliberative methods, and citizens juries specifically in involving the public in decisions making around complex healthcare issues.

<u>Supplementary Note 1 References:</u>

1. Participants' level of trust in public bodies was established using the following question, included in the recruitment questionnaire: 'I will read you a list of different types of people. For each, would you tell me if you generally trust them to tell the truth, or not?' The Scottish Government; local councils; researchers in universities; the NHS; The Police; private companies; charities.
2. Davidson et al (2013), Public Acceptability of Data Sharing Between the Public, Private and Third Sectors for Research Purpose: http://www.gov.scot/Resource/0043/00435458.pdf
