# Supplementary material for: Feasibility and ethics of using data from the Scottish newborn blood spot archive for research
Source: Commun Med (Lond). 2022 Oct 6;2:126. doi: 10.1038/s43856-022-00189-2 (PMC9537278; doi:10.1038/s43856-022-00189-2)
Supplement: Supplementary file 3 — Reporting Summary [file 43856_2022_189_MOESM3_ESM.pdf]

nature portfolio

Corresponding author(s): David Porteous and Sarah Cunningham-Burley

Last updated by author(s): 15/09/2022

Reporting Summary

Nature Portfolio wishes to improve the reproducibility of the work that we publish. This form provides structure for consistency and transparency in reporting. For further information on Nature Portfolio policies, see our Editorial Policies and the Editorial Policy Checklist.

Statistics

For all statistical analyses, confirm that the following items are present in the figure legend, table legend, main text, or Methods section.

n/a	Confirmed	
☐	☒	The exact sample size (*n*) for each experimental group/condition, given as a discrete number and unit of measurement
☒	☐	A statement on whether measurements were taken from distinct samples or whether the same sample was measured repeatedly
☒	☐	The statistical test(s) used AND whether they are one- or two-sided<br>*Only common tests should be described solely by name; describe more complex techniques in the Methods section.*
☒	☐	A description of all covariates tested
☒	☐	A description of any assumptions or corrections, such as tests of normality and adjustment for multiple comparisons
☒	☐	A full description of the statistical parameters including central tendency (e.g. means) or other basic estimates (e.g. regression coefficient) AND variation (e.g. standard deviation) or associated estimates of uncertainty (e.g. confidence intervals)
☒	☐	For null hypothesis testing, the test statistic (e.g. *F*, *t*, *r*) with confidence intervals, effect sizes, degrees of freedom and *P* value noted<br>*Give P values as exact values whenever suitable.*
☒	☐	For Bayesian analysis, information on the choice of priors and Markov chain Monte Carlo settings
☒	☐	For hierarchical and complex designs, identification of the appropriate level for tests and full reporting of outcomes
☒	☐	Estimates of effect sizes (e.g. Cohen's *d*, Pearson's *r*), indicating how they were calculated

Our web collection on statistics for biologists contains articles on many of the points above.

Software and code

Policy information about availability of computer code

Data collection	Excel. No additional software was used.
Data analysis	NA methylation data were processed and analysed in R. Analysis code has been deposited to the following GitHub repository: https://github.com/marioni-group/guthrie. Code from this study, along with version information on the software used, has also been deposited here: doi:10.5281/zenodo.7043056

For manuscripts utilizing custom algorithms or software that are central to the research but not yet described in published literature, software must be made available to editors and reviewers. We strongly encourage code deposition in a community repository (e.g. GitHub). See the Nature Portfolio guidelines for submitting code & software for further information.

Data

Policy information about availability of data

All manuscripts must include a data availability statement. This statement should provide the following information, where applicable:

- Accession codes, unique identifiers, or web links for publicly available datasets
- A description of any restrictions on data availability
- For clinical datasets or third party data, please ensure that the statement adheres to our policy

To access Generation Scotland data, including the data derived in the feasibility study described here, please go to www.ed.ac.uk/generation-scotland/for-researchers/access. Summary-level data analysed in this study has been deposited at https://www.github.com/marioni-group/guthrie

March 2021

Human research participants

Policy information about studies involving human research participants and Sex and Gender in Research.

Reporting on sex and gender	Sex was considered as a factor in juror selection of a representative sample. In the biological analysis, participant's self-reported sex was plotted against biological sex (i.e. based on sex chromosome data) as a means to ensure the correct samples were present.
Population characteristics	Quotas were set to ensure a representative pool of the public in terms of sex, age, working status, and social grade. Additional quotas were set to ensure sufficient representation of people with children under the age of five, and people who had, or whose had a family member with, a pre-existing medical condition. A further, attitudinal quota was set to ensure representation of people with varied levels of trust in public, private and third sector organisations1, as previous research has found this to be a significant factor underpinning views on data sharing and use. Individuals who worked in market research, media, advertising, or journalism, and those who had attended a group discussion or event in the previous 12 months, were excluded from the research.
Recruitment	19 human subjects invited by IPOS Mori to participate in a Citizens Jury following INVOLVER guidelines.
Ethics oversight	The original Sanger DNA sequencing study was mandated by the Scottish Chief Scientist Office, following a favourable opinion from the Scottish Legal Office and Research Ethics Committee. A letter approving the inspection and documentation of Guthrie cards and selective sampling of GS cards for methylation analysis was provided by the Chief Medical Officer for Scotland on 4th September, 2019. The Caldicott Guardians of NHS Greater Glasgow and Clyde and NHS Tayside granted approval on 30th January 2020 and 3rd March 2020, respectively. A substantial amendment to the Research Tissue Bank approval for Generation Scotland to cover the feasibility study was submitted to the Research Ethics Committee and approved on 13th March 2020.

Note that full information on the approval of the study protocol must also be provided in the manuscript.

Field-specific reporting

Please select the one below that is the best fit for your research. If you are not sure, read the appropriate sections before making your selection.

☒ Life sciences ☐ Behavioural & social sciences ☐ Ecological, evolutionary & environmental sciences

For a reference copy of the document with all sections, see nature.com/documents/nr-reporting-summary-flat.pdf

Life sciences study design

All studies must disclose on these points even when the disclosure is negative.

Sample size	Ethics approval was granted for a pilot study of sufficient size and depth to establish feasibility for epidemiolgical and biological research
Data exclusions	There were no data exclusions
Replication	Replication was not possible within the regulatory approval limits of the study
Randomization	There was no randomization as this was an all inclusive pilot feasibility study
Blinding	The study was a feasibility pilot that did not involve blinding. Data linkage was by pseudonomised ID.

Reporting for specific materials, systems and methods

We require information from authors about some types of materials, experimental systems and methods used in many studies. Here, indicate whether each material, system or method listed is relevant to your study. If you are not sure if a list item applies to your research, read the appropriate section before selecting a response.

Materials & experimental systems

n/a	Involved in the study
☒	☐ Antibodies
☒	☐ Eukaryotic cell lines
☒	☐ Palaeontology and archaeology
☒	☐ Animals and other organisms
☐	☒ Clinical data
☒	☐ Dual use research of concern

Methods

n/a	Involved in the study
☒	☐ ChIP-seq
☒	☐ Flow cytometry
☒	☐ MRI-based neuroimaging

Clinical data

Policy information about clinical studies

All manuscripts should comply with the ICMJE guidelines for publication of clinical research and a completed CONSORT checklist must be included with all submissions.

Clinical trial registration	*Provide the trial registration number from ClinicalTrials.gov or an equivalent agency.*
Study protocol	*Note where the full trial protocol can be accessed OR if not available, explain why.*
Data collection	*Describe the settings and locales of data collection, noting the time periods of recruitment and data collection.*
Outcomes	*Describe how you pre-defined primary and secondary outcome measures and how you assessed these measures.*
